# Length Distribution of Ancestral Tracks under a General Admixture Model and Its Applications in Population History Inference

**DOI:** 10.1038/srep20048

**Published:** 2016-01-28

**Authors:** Xumin Ni, Xiong Yang, Wei Guo, Kai Yuan, Ying Zhou, Zhiming Ma, Shuhua Xu

**Affiliations:** 1 Department of Mathematics, School of Science, Beijing Jiaotong University, Beijing 100044, China; 2 Chinese Academy of Sciences (CAS) Key Laboratory of Computational Biology, Max Planck Independent Research Group on Population Genomics, CAS-MPG Partner Institute for Computational Biology (PICB), Shanghai Institutes for Biological Sciences, CAS, Shanghai 200031, China; 3 Institute of Applied Mathematics, Academy of Mathematics and Systems Science, Chinese Academy of Sciences, Beijing 100190, China; 4 School of Life Science and Technology, ShanghaiTech University, Shanghai 200031, China; 5 Collaborative Innovation Center of Genetics and Development, Shanghai 200438, China.

## Abstract

The length of ancestral tracks decays with the passing of generations which can be used to infer population admixture histories. Previous studies have shown the power in recovering the histories of admixed populations via the length distributions of ancestral tracks even under simple models. We believe that the deduction of length distributions under a general model will greatly elevate the power. Here we first deduced the length distributions under a general model and proposed general principles in parameter estimation and model selection with the deduced length distributions. Next, we focused on studying the length distributions and its applications under three typical special cases. Extensive simulations showed that the length distributions of ancestral tracks were well predicted by our theoretical framework. We further developed a new method, *AdmixInfer*, based on the length distributions and good performance was observed when it was applied to infer population histories under the three typical models. Notably, our method was insensitive to demographic history, sample size and threshold to discard short tracks. Finally, good performance was also observed when applied to some real datasets of African Americans, Mexicans and South Asian populations from the HapMap project and the Human Genome Diversity Project.

Population admixture is a common phenomenon in human populations when previously isolated populations start to contact and interact with each other, accompanied by population migration, rising and falling of empires, trading of goods and services, and so on^1^. The history of population admixture does not fade with time, but leaves a great deal of information in the genomes of individuals from admixed populations. Population history in admixed populations thus can be recovered by utilizing the information in the genome, such as break points of recombination^2^, admixture linkage disequilibrium (ALD)^3^,^4^,^5^ and the length of ancestral tracks^1^,^6^,^7^,^8^,^9^,^10^.

The information of ancestral tracks was first used by Pool and Nielsen (which they called migration tracts) to infer population history, and they proposed a model in which a target population received migrants from a source population at a constant rate. The limitation of this model was that gene flow was assumed to be weak enough that any recombination was unlikely between migrant chromosomes^10^. A subsequent study of Pugach *et al*. performed wavelet transform on the ancestral tracks in admixed populations to obtain the dominant frequency of ancestral tracks and compared it to those obtained from extensive simulations, to estimate the admixture time, but under the simple hybrid isolation (HI, or one pulse admixture) model^6^. Then, the study of Jin *et al*. explored admixture dynamics by comparing the empirical and simulated distribution of ancestral tracks under three typical two-way admixture models; i.e. HI model, gradual admixture (GA) model, and continuous gene flow (CGF) model^8^. Jin *et al*. later deduced the theoretical distributions of ancestral tracks under HI and GA models^7^. Gravel extended these studies to multiple ancestral populations and discrete migrations. A numerical estimation of tract length distribution was provided. However, he failed to explicitly deduce the theoretical distribution of ancestral tracks under a general situation^9^.

Here we proposed a general model that can cover all the scenarios of an admixed population with an arbitrary number of ancestral populations and (or) arbitrary number of admixture events. In this study, we first described the general admixture model and deduced a general formula for the theoretical distribution of ancestral tracks with some reasonable approximations. With this distribution, we can use maximum likelihood estimation (MLE) to estimate model parameters, and the Akaike information criterion (AIC)^11^ or the likelihood ratio test (LRT)^12^ to select an optimal model from candidates for the given data. We next demonstrated that the three aforementioned admixture models, namely HI, GA and CGF models in previous studies^8^ are all special cases under our general model. Then, under these three models, we developed a method called *AdmixInfer* to estimate the admixture proportion and admixture time, and simultaneously selected the optimal model according to the principles of AIC. We further conducted extensive simulation studies to demonstrate the accuracy of the theoretical distribution of ancestral tracks under the general model, and the effectiveness of our method to estimate the parameters and select an optimal model. Finally, we applied our method to African Americans and Mexicans from the HapMap phase III dataset^13^, and several South Asian populations from the Human Genome Diversity Project (HGDP)^14^ dataset.

## Methods

### Length distribution of ancestral tracks under a general model

**Figure 1:**
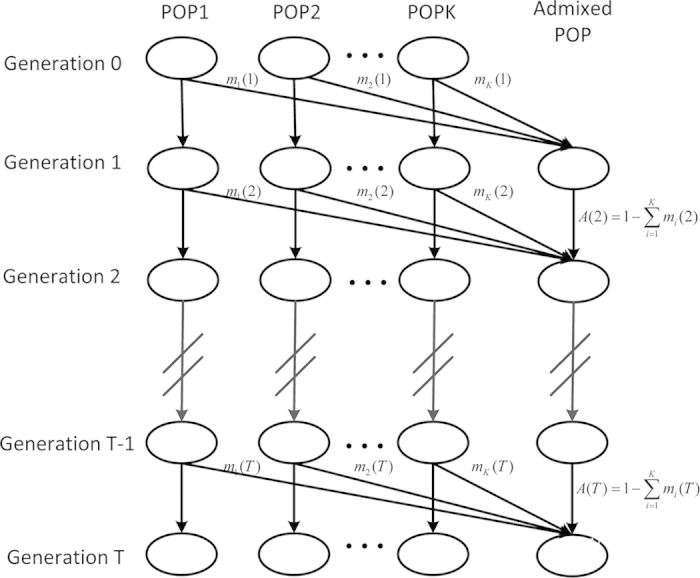
The general admixture model. Here we illustrated an admixed population with *K* ancestral populations, which started to admix *T* generations ago. The gene flows from each ancestral population could be zero at a specific generation. POP *k* represents the ancestral population *k*.

In our general model, population admixture is accomplished by receiving gene flow(s) from ancestral populations either continuously or discontinuously. We model this process generation by generation, in which, if the admixed population does not receive further gene flow(s) in a particular generation, we set the strength of gene flow(s) to 0. Specifically, given an admixed population started *T* generations ago, with *K* ancestral populations, let 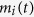 denote the ancestry proportion from the *ith* ancestral population that entered at *t* generation. Here the time of the admixture in generations are denoted to increase over time, with *T* being the present time (see Fig. 1), then the general model is only determined by a *K* × *T* matrix 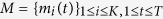, which satisfies three properties: (a) 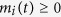; (b) 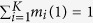 and (c) 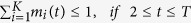.

Let 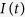 be the ancestry proportion of the admixed population at generation *t* inherited from the previous generation, and then we get 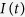 as


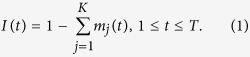


Denote 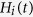 as the total ancestry proportion of the *ith* ancestral population in the admixed population at *t* generation, then


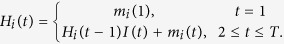


Recursively, we can get


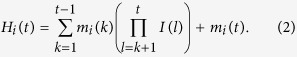


Define 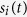 as the survival proportion of the ancestral tracks at generation *T* from the *ith* ancestral population that entered at generation *t*, then


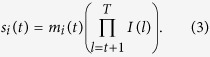


Generally, we assume that the ancestral populations are homogeneous, and recombination among segments from the same ancestry does change the length of the tracks, but it is not “observable” to us, thus the length of tracks seems unchanged, and only these recombination events among different ancestries produce “observable” changes (see Supplementary Fig. S1 online). Here we explicitly take these “unobservable” changes into consideration and adjust the recombination rate accordingly as following. Defining the recombination among tracks from different ancestral populations as effective recombination, let 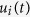 be the effective recombination rate for tracks from the *ith* ancestral population that entered at generation *t*, then


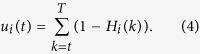


If the end is ignored, a chromosome from the *ith* ancestral population that entered at generation *t* is expected to be split into 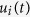 pieces per unit length (unit in Morgan). Then the number of ancestral tracks from *ith* ancestral population that entered at generation *t* to the admixed population is proportional to 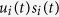. Let 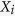 be the length of ancestral tracks of the *ith* ancestral population at generation *T*, and 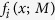 be the distribution of 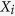, then


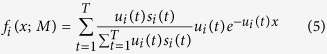


Due to the limited accuracy in local ancestry inference, only those relatively long tracks are reliable^9^,^10^. Therefore, we are also interested in the conditional length distribution of ancestral tracks longer than a specific threshold *C*,


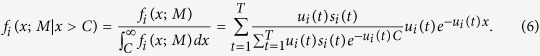


With the length distribution of ancestral tracks (Eq. (5)), we can easily deduce the expectation and variance of 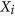 as


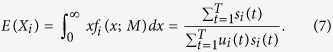


and


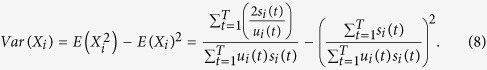


### Parameter estimation and model selection

As the parameters of admixture events are fully determined by the matrix *M*, once *M* is accurately estimated, we can fully recover the history of the admixed population. MLE can be used to obtain the estimation of *M*. By utilizing the ancestral tracks inferred from the data, a likelihood function can be computed with the length distributions of ancestral tracks. The log-likelihood function of ancestral tracks from the *ith* ancestral population 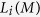 has the form of


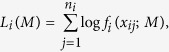


where 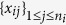 are the observed lengths of tracks from the *ith* ancestral population in the admixed population. Then the log-likelihood function of ancestral tracks of the admixed population 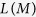 is


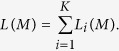


Then the estimator of *M* is


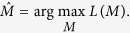


where *M* satisfies the properties in the above subsection.

However, with the increase in the number of parameters, it is complex and time-consuming to find the optimal solution and too many parameters can lead to over-fitting. In a real situation, we can propose several candidate models with prior knowledge in which the number of parameters is dramatically reduced, thus the problem is simplified to estimating parameters for each candidate model and selecting the most suitable one. If we have obtained the parameters of the candidate models, we can compare the models in a pair-wise fashion by using either AIC or LRT. Here, the two models are regarded to be ‘nested’ if one of the models constitutes a special case of the other^15^. When the two competing models are nested, we use LRT to select the model; otherwise we use AIC.

### The distribution of ancestral tracks under HI, GA, and CGF Models

In this subsection, we demonstrate that, with the length distribution of the general model, we can easily deduce the length distributions of ancestral tracks under three typical models aforementioned in previous studies^8^. By restricting the number of ancestral populations to be two in the general model, if only one pulse of gene flow is allowed, the model turns into HI model; if extra equal gene flows from both ancestral populations are allowed, the model turns into GA model; if extra equal gene flows from only one of the ancestral populations are allowed, the model turns into CGF model (see Supplementary Fig. S2 online). Thus these three models are all special cases of our general model. There are only two parameters in each of the three models: the admixture proportion *m* and the admixture time *T*. Easily we can obtain the distribution of the ancestral tracks of these three models from Equation (5). Detailed calculation is in Supplementary Text S1 online.

For simplicity, we define the ancestral population with the minor ancestry contribution as population 1, and the corresponding proportion is *m*. For HI model (see Supplementary Fig. S2 online), the ancestry proportions from population 1 and population 2 at generation *t* are


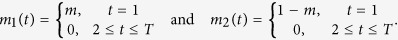


Then the length distribution of ancestral tracks from population 1 is


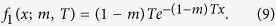


We can also get the expectation and variance of the length of ancestral tracks from Equation (7) and Equation (8),


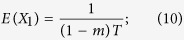



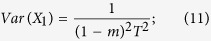


Substituting *m* with 1*−m* in Equations (9), (10) and (11), we can obtain the length distribution, expectation, and variance of ancestral tracks from population 2, respectively. These two distributions are identical to the ones in previous studies^7^,^9^.

For GA model (see Supplementary Fig. S2 online), the ancestry proportions from population 1 and population 2 at generation *t* are


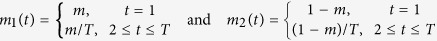


Then the length distribution of ancestral tracks from population 1 is


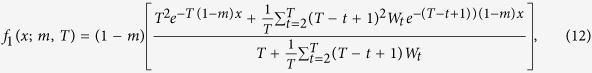


where 
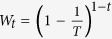
. The expectation and variance of the length of ancestral tracks are


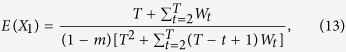



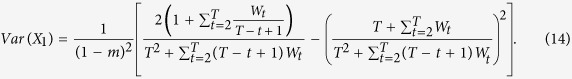


Substituting *m* with 1−*m* in Equations (12), (13), and (14), we can get the length distribution, expectation and variance of ancestral tracks from population 2, respectively.

For CGF model (see Supplementary Fig. S2 online), the ancestral population that contributes only one pulse of gene flow is treated as a gene flow recipient and the one that contributes continuously as gene flow donor. Here, we divide the CGF model into two sub-models. If population 1 is a gene flow recipient, we denote it as CGFR model; otherwise we denote it as CGFD model.

In the case of CGFR model, the ancestry proportions from population 1 and population 2 at *t* generation are


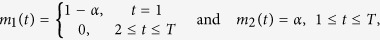


where 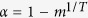. Then the length distributions of ancestral tracks from the two ancestral populations are


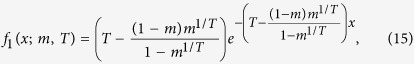



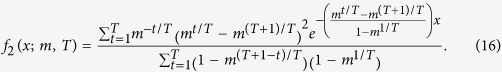


The expectations and variances of the length of ancestral tracks are


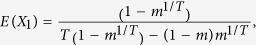



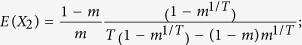



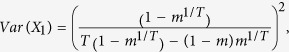



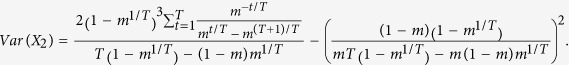


In the case of CGFD model, we just replace *m* with 1−*m* in Equation (15) and (16), and obtain the length distribution of ancestral tracks from population 2 and population 1, respectively.

### Parameter estimation and model selection under HI, GA, and CGF models

As discussed above, there are only two parameters *m* and *T* in HI, GA and CGF models. As for *m*, with the inferred ancestral tracks in an admixed population, we divide the total length of tracks from population 1 by the total length of tracks, and obtain an estimator 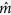 of the admixture proportion. Interestingly, from the expectation of the ancestral tracks from two ancestral populations in HI, GA and CGF models, we find that the expectation ratio between population 1 and population 2 relies only on *m*,


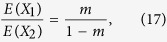


thus we provide an alternative way to obtain the estimator 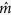,


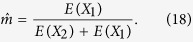


Generally, if there are only two ancestral populations, Equation (17) always holds whatever the admixture model is. The proof is in Supplementary Text S1 online.

As for admixture time *T*, the estimation relies on the model assumed. Different models give different estimations of *T* so that we first need to assume a model. Here we regard HI, GA, CGFR and CGFD models as the candidate models, and use MLE to estimate the admixture time *T* and AIC to select the optimal model as following: First, by utilizing the inferred ancestral tracks, we calculate the admixture proportion and determine population 1; secondly, with the estimator 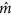, maximizing the likelihood under model assumption HI, GA, CGFR, and CGFD, we obtain the maximum likelihoods 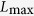 (HI), 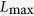 (GA), 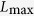 (CGFR) and 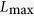 (CGFD) and corresponding optimal times 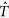. Because each pair of these models is not nested, thus here we use AIC to select the optimal model. The value of AIC can be calculated by the equation


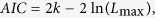


where *k* is the number of parameters and 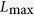 is the maximized value of the likelihood function. The number of parameter of these models are the same, thus at the end of the comparison, we find that the problem is equivalent to finding the model with the highest likelihood. Thus, the model with the highest likelihood is chosen as the optimal model, and the corresponding parameters as the final results. These routines are implemented in our *AdmixInfer*. We also apply the bootstrapping technique in *AdmixInfer* and give a bootstrapped supporting rate of the chosen model and the estimation and confidence interval (CI) of the admixture time (Details in Supplementary Text S1 online).

### General settings of simulation studies

Simulation studies were performed to evaluate the correctness of the length distribution of ancestral tracks under the general model, and the performance of *AdmixInfer* under three typical models. The following settings remained the same under all situations simulated if with no further modification: The population size of the admixed population was simply set to 5,000 and remained constant in our simulations and the length of simulated chromosome was 3.0 Morgan, which approximated the length of chromosome 1 of the human genome. We simulated one chromosome each time and a pair of chromosomes represented an “individual.” At the end of simulation, 400 “individuals” (genome length of an “individual” approximated 1/10 of the length of a human individual) were sampled and the ancestral tracks were directly recorded. Simulations were carried out by a forward-time simulator. *AdmixSim* simulates admixture processes with more than two parental populations, a given number of admixture events, and fluctuated population sizes across generations under Wright Fisher model.

### Evaluate the theoretical distribution under the general model

To test the accuracy of the length distribution of ancestral tracks under the general model, we simulated several general representative cases with three or four ancestral populations and one or more waves of admixture. Simulation 1A was to simulate one pulse admixture with three ancestral populations: admixture started 50 generations ago with proportions 10%, 30% and 60%, respectively. Simulation 1B was to simulate discrete multiple-waves admixture with three ancestral populations: the admixture started 50 generations ago with initial proportions 10%, 40% and 50%, respectively, and population 1 contributed extra 10% ancestries each 10 generations later. Simulation 1C was to simulate continuous multiple-waves admixture with three ancestral populations: the admixture started 50 generations ago with initial proportions 10%, 30% and 60%, respectively; population 1 contributed an extra 0.2% ancestries for each generation afterwards. And simulation 1D was to simulate multiple-waves admixture with four ancestral populations that arrived at different times: populations 1 and 2 firstly admixed 50 generations ago with proportions 40% and 60%, population 3 entered 40 generations ago with proportion 20%, and population 4 entered 30 generations ago with proportion 10%. The simulations were repeated 5 times, and the ancestral tracks in the admixed population were recorded and the length distribution was compared to the theoretical distribution. Detailed parameters for the simulations were provided in Supplementary Data S1 online.

### Evaluate the performance of *AdmixInfer*

We focused on evaluating the performance of *AdmixInfer* under three typical models; i.e. HI, GA and CGF. Admixture proportions varied from 10% to 50% in steps of 10% for the admixture models (HI, GA, CGFR and CGFD). We set the admixture time as 5, 10, 20, 30, …, 200 generations. The ancestral tracks in admixed populations were also recorded as previous simulations. Each simulation here was repeated 10 times and, in total, 4,200 simulations were carried out under HI, GA and CGF models. *AdmixInfer* was applied to the simulated data with the default settings; the results were recorded and summarized.

In real situations, we could only accurately infer the ancestral tracks longer than a specific threshold due to methods’ limitations in local ancestry inferences. To make our method more feasible to real cases, we also evaluated the robustness of our method under different thresholds ranging from 0 centi-Morgan (cM) to 2 cM in step of 0.1 cM, with the dataset simulated in previous evaluations.

We also evaluated the performance of *AdmixInfer* with different sample sizes. We simulated populations starting with the admixture of 50 and 100 generations ago under HI, GA, and CGF models, with admixture proportions 30%:70%. At the end of the simulation, 10, 20, 50, 100, 200, and 500 “individuals” were sampled, corresponding to 1, 2, 5, 10, 20, and 50 human samples. *AdmixInfer* was applied to the simulated dataset without discarding short tracks.

Finally, we tested the performance of our method with data simulated by real populations and inferred ancestral tracks. Simulations were carried out with real populations YRI and CEU as parental populations under different models (30% YRI ancestry and 70% CEU ancestry) with admixture time 10, 20, 50 and 100 generations. Here we simulated with the data of chromosome 1 and sampled 25 “individuals” at the end of the simulation and each simulation was repeated 10 times. Then the local ancestry of the simulated populations was inferred by HAPMIX^16^. With the derived ancestral tracks, *AdmixInfer* was used to select the optimal model and estimate generations accordingly with the tracks longer than 1 cM.

### Apply to real datasets

We applied our method to some real datasets. First, the histories of African Americans and Mexicans are relatively clear, thus they can be used to test the performance of our method. The data of African Americans, Mexicans and reference populations CEU and YRI were obtained from HapMap project phase III^13^, and the reference populations that represented American Indian ancestry (Maya and Pima) were obtained from HGDP dataset. Although previous studies showed multi-ancestry of African Americans and Mexicans^7^,^17^, here we mainly focused on the two dominant ancestries. Then we also applied our method to several HGDP populations from South Asia^14^, which showed evidence of population admixture from previous studies^1^,^3^. Haplotype phasing was performed by SHAPEIT 2^18^. Local ancestry was inferred by HAPMIX^16^. According to the prior knowledge, the generations set in HAPMIX inference were 10, 20 and 50 for African Americans, Mexicans and South Asian populations, respectively^1^. *AdmixInfer* was used to select the optimal model and admixture time accordingly with the tracks longer than 1 cM. We also performed bootstrapping 100 times to obtain confidence of model selection, and calculated the 95% confidence intervals of the generations inferred.

**Data availability**: The source codes and tutorials of *AdmixInfer* and *AdmixSim* are available at: http://www.picb.ac.cn/PGG/resource.php.

## Results

### Theoretical and simulated distributions of ancestral tracks match well

**Figure 2:**
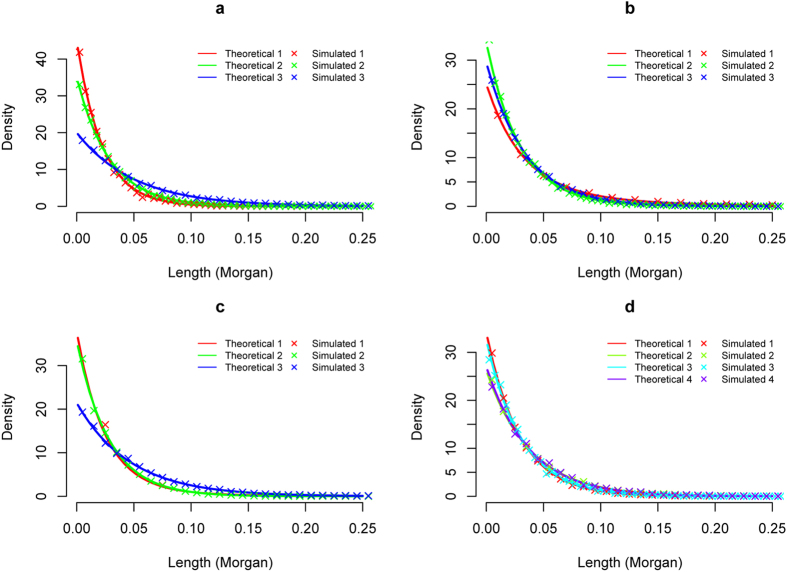
Theoretical and simulated distributions of ancestral tracks under some representative admixture scenarios. **(a)** three reference populations admixed once at 50 generations ago; **(b)** three reference populations admixed at 50 generations ago and the first population contributed an extra 10% ancestry each 10 generations later; **(c)** three reference populations admixed at 50 generations ago and the first population contributed an extra 0.2% ancestry every generation later; **(d)** two reference populations admixed 50 generations ago, the third reference population contributed 20% ancestry 40 generations ago, and the fourth reference population contributed 10% ancestry 30 generations ago.

With the length distributions of ancestral tracks under the general model, we could easily sketch the curves of theoretical length distributions of ancestral tracks under a given model (see Fig. 2). By putting the theoretical and simulated length distributions of ancestral tracks together, we clearly observed that the theoretical and simulated distributions of ancestral tracks matched well, for all the situations simulated and all the replicates (see Fig. 2). It showed that the theoretical length distribution of ancestral tracks for the general model, which we deduced, was reasonable and accurate.

### *AdmixInfer* performs well in parameters estimation and model selection

With the extensively simulated data, we could systematically evaluate the performance of our method in parameters estimation and model selection. For the simulated admixed populations under HI model or CGFD model, our method could always distinguish the right model in all our simulations; for the CGFR model, our method could distinguish the right model with accuracy of 97.0%; and for GA model, our method could distinguish the right model with accuracy of 93.0% (see Table 1). Moreover, the specificity of our method was over 97% for all the situations simulated. The sensitivity of GA and CGFR models and the specificity of HI and CGFD models under different admixture proportions and different admixture times were shown in Supplementary Fig. S3 online. We found that the simulations in which our method could not distinguish the right ones were mostly observed in these simulations with very recent admixture times and small admixture proportions (see Supplementary Fig. S3 and Data S2–S6 online). We also found that almost all CGFR models were only wrongly distinguished as HI models, and GA models as CGFD models (see Supplementary Data S6 online). This is also reasonable, because CGFR model is close to HI model, so that CGFR model is more likely to be distinguished as HI model. The same reason applies to GA model being wrongly distinguished as CGFD model.

**Figure 3:**
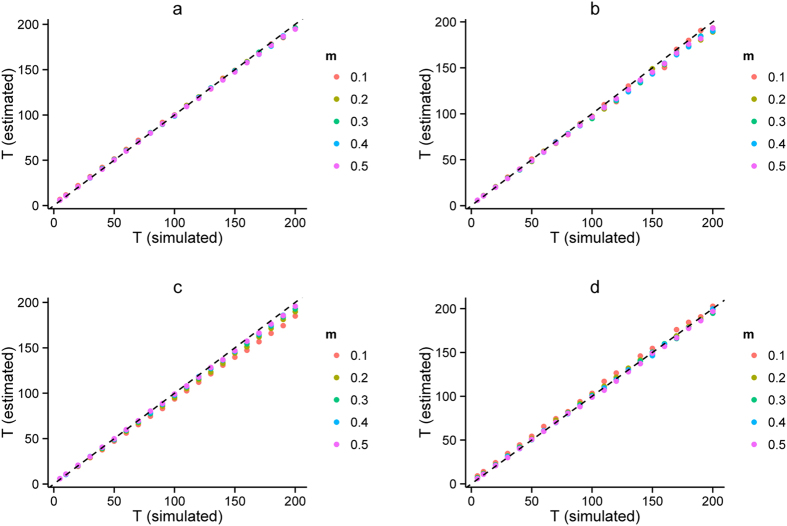
Mean generations estimated from simulation. Each dot denotes the mean generation of ten simulation replicates. **(a)** mean generations estimated under HI model; **(b)** mean generations estimated under GA model; **(c)** mean generations estimated under CGF model (population 1 as gene flow recipient); and **(d)** mean generations estimated under CGF model (population 1 as gene flow donor). Different colors represent different simulated proportions of population one.

Note that there were only two parameters *m* and *T* for the three typical models. Our method also performed well in estimating parameters *m* and *T* for the three typical models. Regarding the admixture proportion *m*, estimations were very close to the pre-settings in simulations, and the small deviation was due to random drift in simulation with finite population sizes and only a subset of individuals being sampled at the end of the simulation. Time estimations for wrongly distinguished models were meaningless, and thus should be discarded. Results showed that our method can estimate admixture times with high accuracy (see Fig. 3, Supplementary Fig. S4–S7, and Data S2–S5). For HI and CGFD models, results showed high consistency with the time simulated, while a slight underestimation occurred for GA and CGFR models. For all these models that were simulated, the time estimated for a small proportion was less accurate than that for a larger proportion. We defined the relative errors between true admixture times and inferred admixture times as


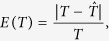


where *T* is the true admixture time and 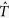 is the estimation of the admixture time. Under the situation of a certain admixture proportion and a certain model, we defined the average relative error 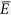 on different values of admixture time as


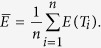


We found that when the admixture proportion was 0.1, the relative errors of CGFR and CGFD were 6.43% and 5.89%, respectively. For other cases, the relative errors were all less than 4% (see Table 2). In conclusion, no matter for the model selection or parameters estimation, our method performed well.

### Robustness for different thresholds for length of ancestral track

**Figure 4:**
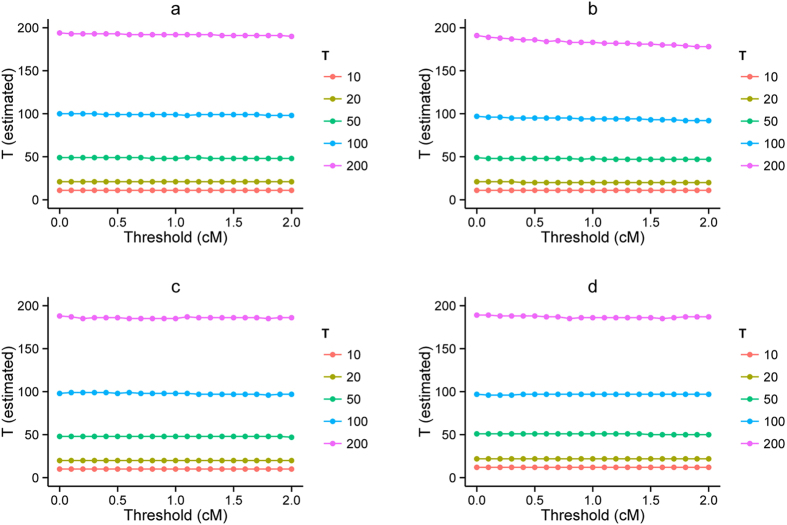
Generation estimated with different thresholds from simulation. Models simulated are **(a)** HI, **(b)** GA, **(c)** CGFR and **(d)** CGFD. The simulated admixture proportion was 30%. Different colors represent different simulated generations.

To test the robustness of our method for different thresholds for length of ancestral track, we tested our method under different thresholds varying from 0 cM to 2 cM in steps of 0.1 cM. The results showed that our method was robust to thresholds, except GA model with a larger time (see Fig. 4). When a larger threshold is taken, less information is kept for ancient admixture events. Although it is better to keep all the information to estimate admixture times, we must balance the trade-off between information and accuracy, because the accuracy of local ancestry inference is not so good for short ancestral tracks due to method limitations. Take HI for an example, the probability *p* of ancestral tracks larger than a specific threshold *C* is


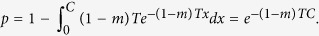


Therefore, with an increase in threshold *C*, less information is kept. Here, we provided a reference table of the probability *p* under different admixture times and proportions (see Supplementary Data S7 online). For example, when *T* = 200, *m* = 0.1, if we set the threshold *C* as 2 cM, the probability that the tracks exceed *C* is only 2.7%.

### Small sample sizes also give good estimations

**Figure 5:**
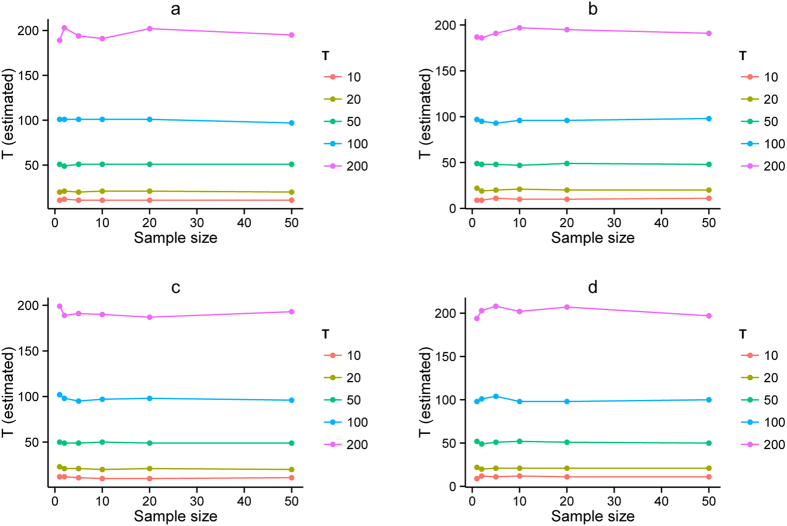
Admixture time in generations estimated with different sample sizes. Models simulated are **(a)** HI, **(b)** GA, **(c)** CGFR and **(d)** CGFD. Different colors represent different simulated generations. The simulated admixture proportion was 30%.

To test the performance of *AdmixInfer* with different sample sizes, we evaluated the models with 10, 20, 50, 100, 200, and 500 “individuals” (corresponding to 1, 2, 5, 10, 20, and 50 human samples). Results showed that *AdmixInfer* was insensitive to sample sizes. Even with only one human sample, it could distinguish the right model and estimate the admixture time with high accuracy (see Fig. 5). However, considering the accuracy of local ancestry, short tracks were usually discarded. The information kept for extremely small sample sizes might not be sufficient to give a clear picture of the history of a population. Therefore, relatively larger sample sizes were recommended.

### Error analysis

When we use our method to infer the history of a real admixed population, there are two kinds of errors that may influence the accuracy of inference. The first kind of error is caused by the assumptions in deducing the length distribution of ancestral tracks. In the derivation, for simplicity, we ignored the end of the chromosome and the drift. For this kind of error, we have used the simulation data to demonstrate that the accuracy of the inference was neglectable (see Fig. 3 and Table 1). The second kind of error is caused by the local ancestry inference. In our study, local ancestral tracks are inferred by HAPMIX software. Here we used the simulation data with ancestry populations YRI and CEU to analyze the influence of this kind of error. For all the cases we simulated, only HI model with an admixture time 100 generations was wrongly distinguished as GA model (see Supplementary Table S1 online). We also found that in the case of large admixture times, the error of local ancestry inference will cause underestimation of admixture time. When the admixture time is large, the ancestral tracks will be short. However, the method of inferring ancestral tracks cannot effectively determine short tracks. Thus, it will influence the accuracy of our method in inferring admixture times and model selections.

### Real data analysis

Our method for parameters estimation and model selection under three typical models implemented in *AdmixInfer* was applied to a real dataset. First, African American was inferred as GA model and the admixture time was 12 generations ago (see Table 3). When 29 years per generation was assumed according to previous investigation^19^, it was about 350 years ago, which was consistent with the recorded history that most African ancestors arrived in America (via slave trading) after the seventeenth century. The slave trade continued until the nineteenth century and after that, many African people settled down in America. Gene flows from Africa and Europe would have continuously contributed to the African American gene pool and thus GA model matched the recorded history well. Similarly, the model for Mexicans was also inferred as GA model and the admixture time was 18 generations ago (522 years before present), which was also consistent with the time of the exploration of the new world and the arrival of Europeans. GA model indicates continuous contact and admixture of Europeans and American Indians. Moreover, all these inferences are close to previous findings^17^.

Finally, we studied the admixture histories of several HGDP populations from South Asia. Previous studies have shown that the populations from South Asia have admixed ancestries mainly from Europe and East Asia^1^,^14^. Regarding the admixture proportions, our estimations based on *AdmixInfer* (see Table 3) were consistent with previous estimations from the *f*-statistics^3^. Regarding the admixture model and time, the populations Balochi, Brahui, Burusho, Kalash, Pathan and Sindhi from Pakistan in South Asia were all inferred to as CGF (Sardinian as donor), which indicated extra gene flows from European ancestry after initial admixture. The initial admixture times estimated ranged from 107 to 96 generations ago. When 29 years per generation was assumed according to previous investigations^19^, these estimations (1103BC-784BC) coincided with the migration of Indo-Aryan speaking people into the Indian subcontinent. Extra gene flows from European ancestry might be contributed to by the rising of empires during the following centuries. In the case of the populations of Hazara and Uygur, they not only showed very similar admixture proportions, but also showed the same admixture model and very close admixture times; i.e., 67 and 70 generations ago, respectively. The Hazara population mainly settled in Afghanistan and Pakistan, while the Uygur population mainly settled in West China, and both populations are connected along the Silk Road. It was feasible to receive continuous gene flows from both European and East Asian ancestries. These similarities also indicated a possible close relationship or shared histories between these two populations.

In summary, our method showed good performance in inferring the admixture history of African Americans and Mexicans. The admixture scenarios in South Asian populations were more complex than expected. However, with our method, the analysis could shed light on the mysterious histories of these populations.

## Discussion

In this work, we proposed a general model to describe the admixture history with multiple ancestral source populations and multiple-wave admixtures. We showed the length distribution of ancestral tracks and some of its useful properties under this general model. We thus provided a theoretical framework to study population admixture history. With the general framework, we focused on studying three special cases of admixture models (HI, GA, and CGF) and developed a method to estimate admixture proportion, admixture time and determine optimal model simultaneously. Our simulations showed that the theoretical distribution of ancestral tracks was consistent with our theoretical prediction, and our method was precise and efficient in inferring population history under the three typical models.

Comparing with the method of Gravel^9^, we deduced the length distribution of ancestral tracks without drift and assumed infinite chromosome length, and found that the length distribution was a mixed exponential distributions. Simulations demonstrated that these approximations were reasonable and accurate when admixture time *T* was not too small. In the case of *T* was too small, the length distribution did not follow an exponential distribution, as pointed out by Liang and Nielsen^20^, which could also explain the poor performance of our methods for small *T* (e.g. 5 generations).

In the efforts of model selection, we found that the simulations in which our method was not able to determine the correct model, were mostly those cases with recent admixture times and minor admixture proportions. The possible reason for incorrect determination was that we ignored the chromosome ends in deducing the theoretical length distribution. When the admixture proportion and times were small, the chromosomes without “observable” recombination were over-represented in the ancestral tracks (see Supplementary Fig. S8 online). Our further simulations showed that when the chromosome length increased, the accuracy of our method was enhanced. Furthermore, we note that the length distributions of ancestral tracks have no relationship with the population size. Thus, the change of population size does not affect the time estimation, which is also supported by simulations under different demographic models (see Supplementary Fig. S9 online).

The efficiency of our method could also be influenced by the validity of the local ancestry inference. To improve the reliability of the inference, we suggest using the ancestral tracks longer than a certain threshold *C*. However, when the threshold became large, some ancient admixture information disappeared rapidly. In principle, if short ancestral tracks could be precisely detected, our method is promising in recovering even more ancient admixture history, such as the admixture between modern human and Neanderthals^21^,^22^.

Though we proposed a general framework and relevant principles to infer the population history under the general model, finding optimal estimation for parameters is challenging work with high dimensionality. Currently, our method implemented in *AdmixInfer* is focusing on the three typical models. For the real admixed populations, the admixture history is always complex, such as discrete multiple-waves admixture. Under such circumstances, the length distribution of ancestral tracks under the general model is still broadly useful and applicable. Therefore, based on this framework, to infer more complicated admixture history is a problem to be solved in the future.

## Additional Information

**How to cite this article**: Ni, X. *et al*. Length Distribution of Ancestral Tracks under a General Admixture Model and Its Applications in Population History Inference. *Sci. Rep.* **6**, 20048; doi: 10.1038/srep20048 (2016).

## Supplementary Material

Supplementary Information

Supplementary Dataset

## Figures and Tables

**Table 1:** The accuracy of our methods in model selection under three typical models.

**Model**	**TP**	**FN**	**TN**	**FP**	**Sensitivity (%)**	**Specificity (%)**
HI	1050	0	3120	30	100.0	99.0
GA	977	73	3150	0	93.0	100.0
CGFR	1019	31	3150	0	97.0	100.0
CGFD	1050	0	3076	74	100.0	97.7

HI: Hybrid isolation model; GA: Gradual admixture model; CGFR: Continuous gene flow model (population 1 as recipient); CGFD: Continuous gene flow model (population 1 as donor). TP: True positive; FP: False positive; TN: True negative; FN: False negative. Sensitivity = TP/(TP + FN); Specificity = TN/(TN + FP).

**Table 2:** The average relative error 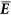 for different models and different values of admixture proportions.

***m***	**HI**	**GA**	**CGFR**	**CGFD**
0.1	0.02304	0.02245	0.06433	0.05893
0.2	0.01327	0.03171	0.03917	0.02396
0.3	0.01149	0.02939	0.02439	0.01629
0.4	0.01260	0.02969	0.01704	0.01386
0.5	0.01158	0.02547	0.01474	0.01475

*m*: Admixture proportion; 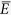: Average relative error on different values of admixture times. Here we discard the cases of 5 and 10 generations admixture time because wrongly distinguished models mainly focused on these two admixture times.

**Table 3:** Admixture time and model inferred for real datasets.

**POP1**	**POP2**	**Admixed pop**	***m***	**Model**	***T***	**95% CI**
CEU	YRI	ASW	0.247	GA (99%)	12	[12,12]
CEU	AMI	MEX	0.478	GA (100%)	18.02	[17.99, 18.05]
JAP	SAR	Uygur	0.570	GA (100%)	70.43	[70.2, 70.66]
JAP	SAR	Hazara	0.559	GA (100%)	67.16	[67.01, 67.31]
JAP	SAR	Balochi	0.198	CGF^*^ (100%)	104.7	[104.5, 104.8]
JAP	SAR	Brahui	0.190	CGF^*^ (100%)	105.2	[105, 105.3]
JAP	SAR	Burusho	0.339	CGF^*^ (100%)	96.52	[96.34, 96.7]
JAP	SAR	Kalash	0.174	CGF^*^ (100%)	100.5	[99.27, 101.6]
JAP	SAR	Pathan	0.258	CGF^*^ (100%)	98.49	[98.28, 98.7]
JAP	SAR	Sindhi	0.272	CGF^*^ (100%)	107.5	[107.4, 107.7]

POP1: Reference population one; POP2: Reference population two; Admixed pop: Admixed population; *m*: Admixture proportion of reference population one; Model: Inferred admixture model, percentage in the parenthesis is the support rate in 100 times bootstrapping; *T*: estimated admixture time in generation; 95%CI: 95% confidence interval of the estimated admixture time; AMI: Combined dataset of populations Maya and Pima which represent American Indian ancestry; JAP: Japanese; SAR: Sardinian; * Sardinian as donor population.
