# Corrigendum: Length Distribution of Ancestral Tracks under a General Admixture Model and Its Applications in Population History Inference

**DOI:** 10.1038/srep26367

**Published:** 2016-05-23

**Authors:** Xumin Ni, Xiong Yang, Wei Guo, Kai Yuan, Ying Zhou, Zhiming Ma, Shuhua Xu

Scientific Reports 6: Article number: 2004810.1038/srep20048; published online: 01282016; updated: 05232016.

In the original version of this Article, Affiliation 1 was omitted for Zhiming Ma. This error has now been corrected in the PDF and HTML versions of the Article.

