# Supplementary material for: Length Distribution of Ancestral Tracks under a General Admixture Model and Its Applications in Population History Inference
Source: Sci Rep. 2016 Jan 28;6:20048. doi: 10.1038/srep20048 (PMC4730239; doi:10.1038/srep20048)
Supplement: Supplementary Information [file srep20048-s1.pdf]

Supplementary Information for

Length Distribution of Ancestral Tracks under a General Admixture Model and Its Applications in Population History Inference

Xumin Ni[+], Xiong Yang [+], Wei Guo, Kai Yuan, Ying Zhou, Zhiming Ma[*], Shuhua Xu[*]

[*]Corresponding author. E-mail: xushua@picb.ac.cn (S.X.) and mazm@amt.ac.cn (Z.M.). [+]These authors contributed equally to this work.

Supplementary Figure S1

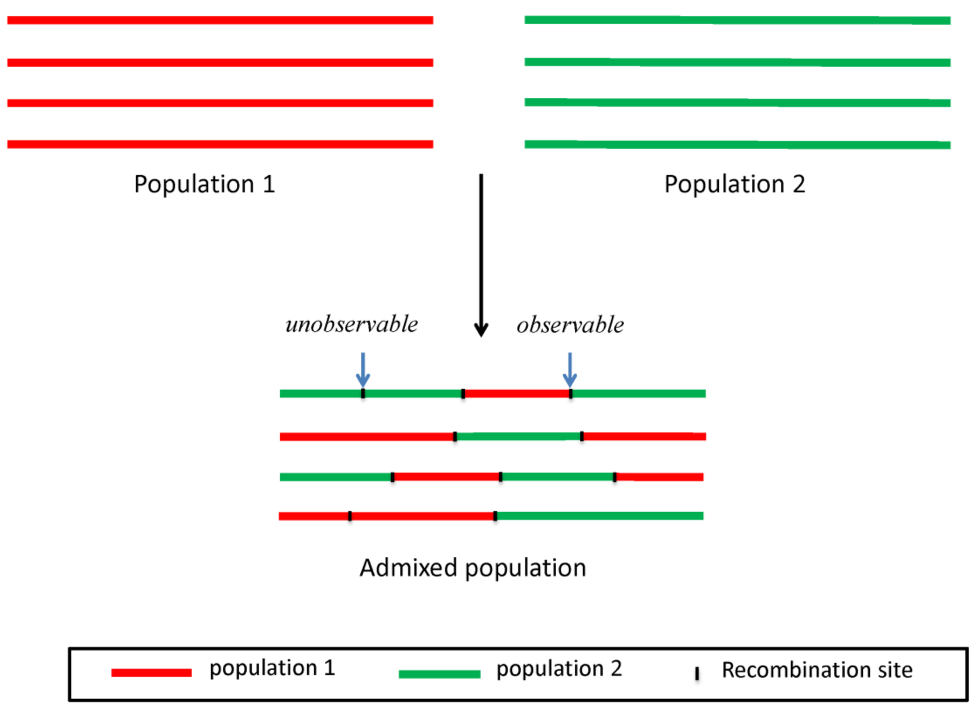

Fig. S1. Two types of recombination: observable and unobservable recombination. Recombination among segments from the same ancestry is unobservable recombination, otherwise is observable recombination.

Supplementary Figure S2

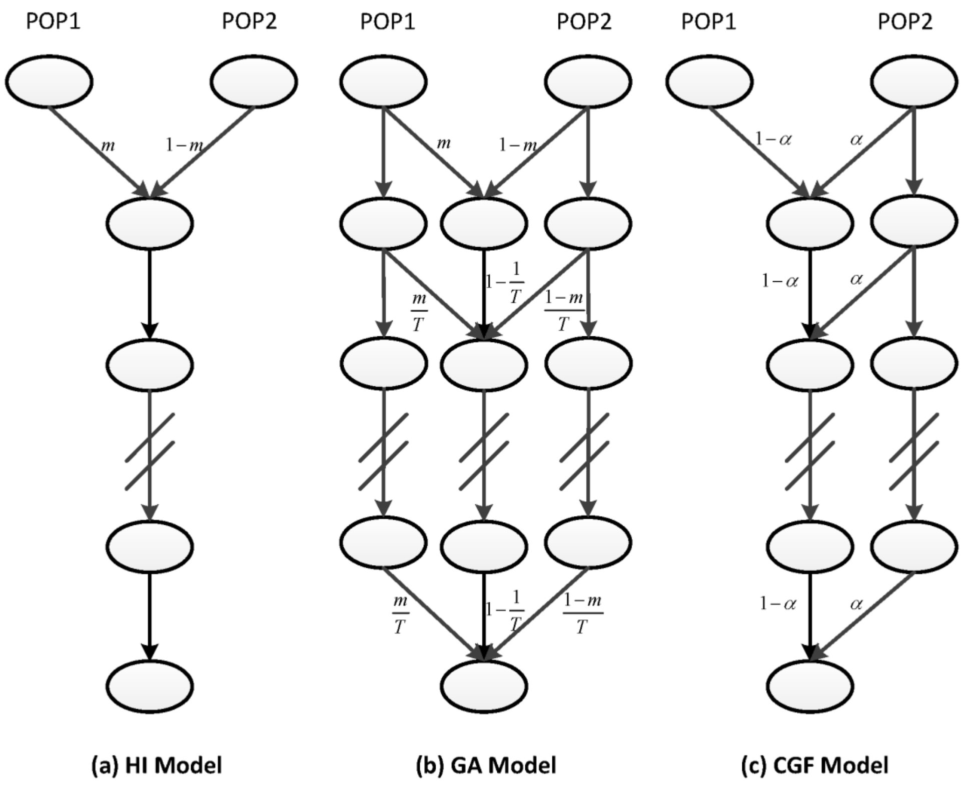

Fig. S2. **Three typical admixture models.** **(a)**: Hybrid isolation (HI) model; **(b)**: Gradual admixture (GA) model; **(c)**: Continuous gene flow (CGF) model. POP1: the reference population one; POP2: the reference population two; m is proportion of population one and $\alpha = 1 - m^{1/T}$.

Supplementary Figure S3

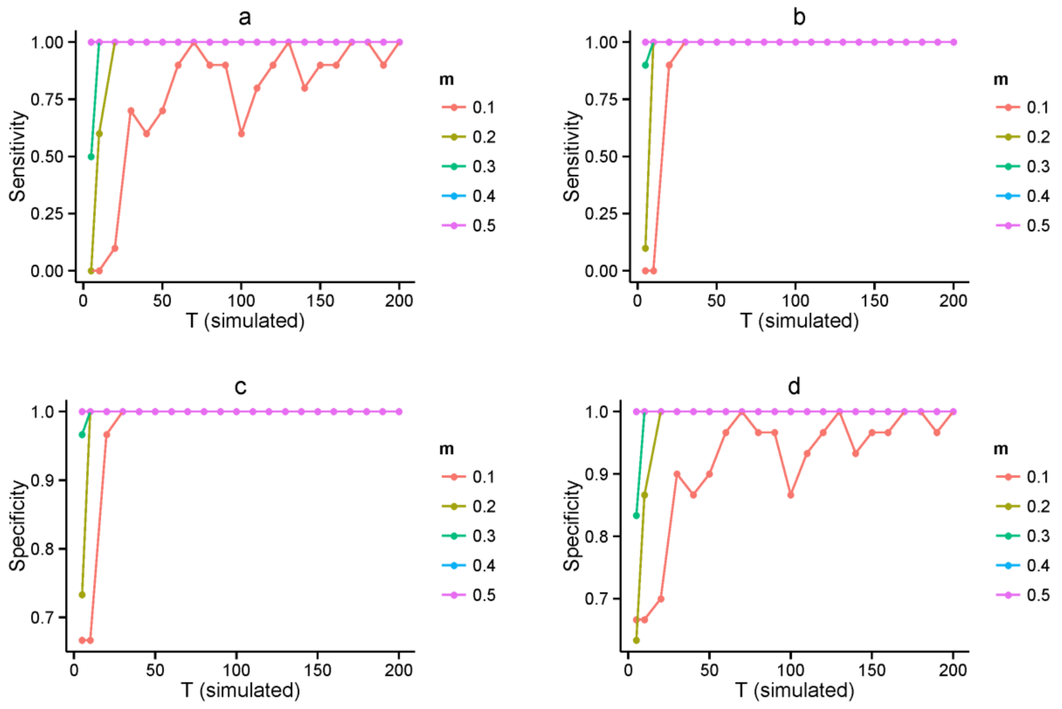

Fig. S3. **The sensitivity and specificity of model selection.** (**a)**: Sensitivity of model selection for GA model; **(b)**: Sensitivity of model selection for CGFR model; **(c)**: Specificity of model selection for HI model; **(d)**: Specificity of model selection for CGFD model. Different colors represent different simulated proportions of population one.

Supplementary Figure S4

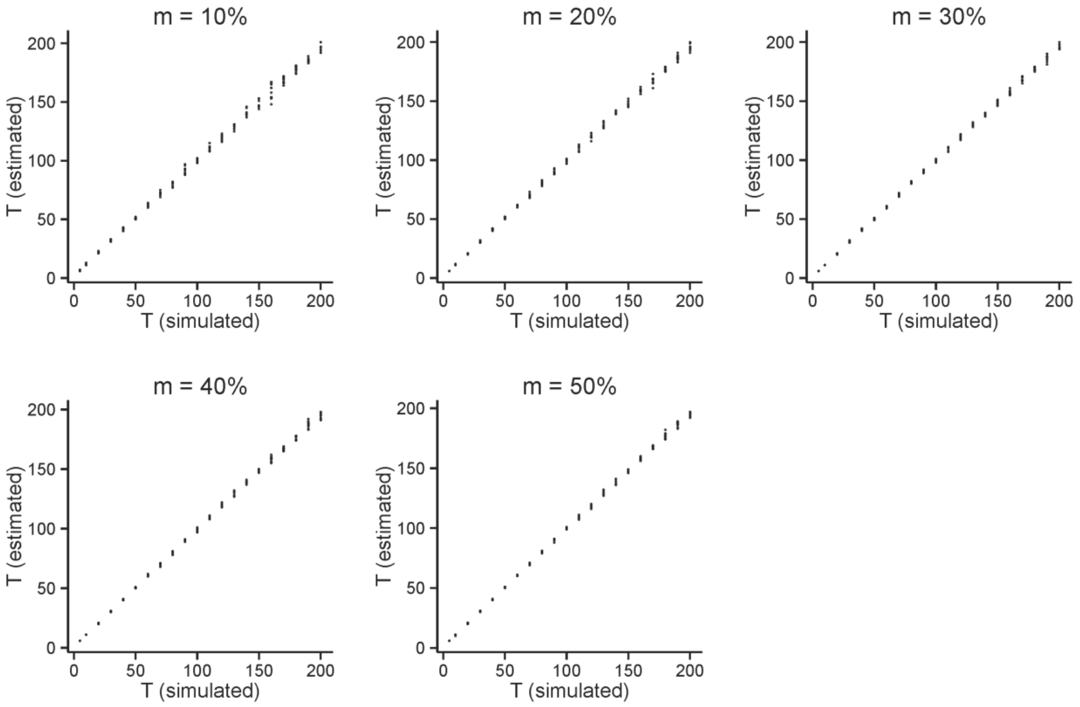

Fig. S4. **Generation estimated for each simulation replicate under HI model.**

Supplementary Figure S5

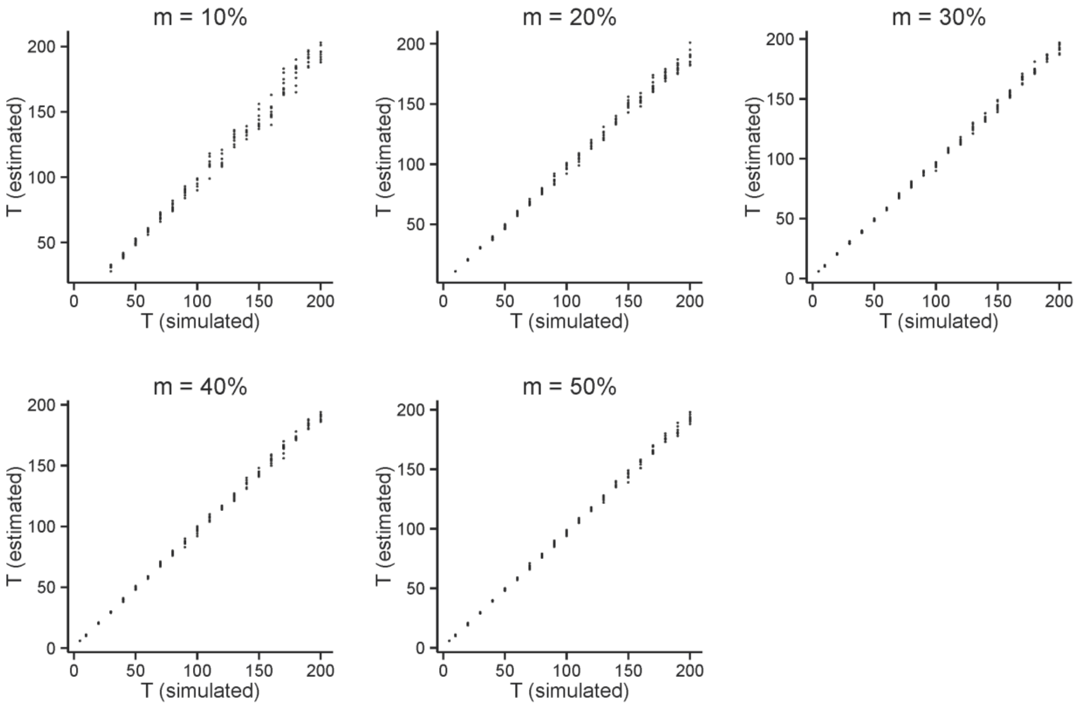

Fig. S5. **Generation estimated for each simulation replicate under GA model.**

Supplementary Figure S6

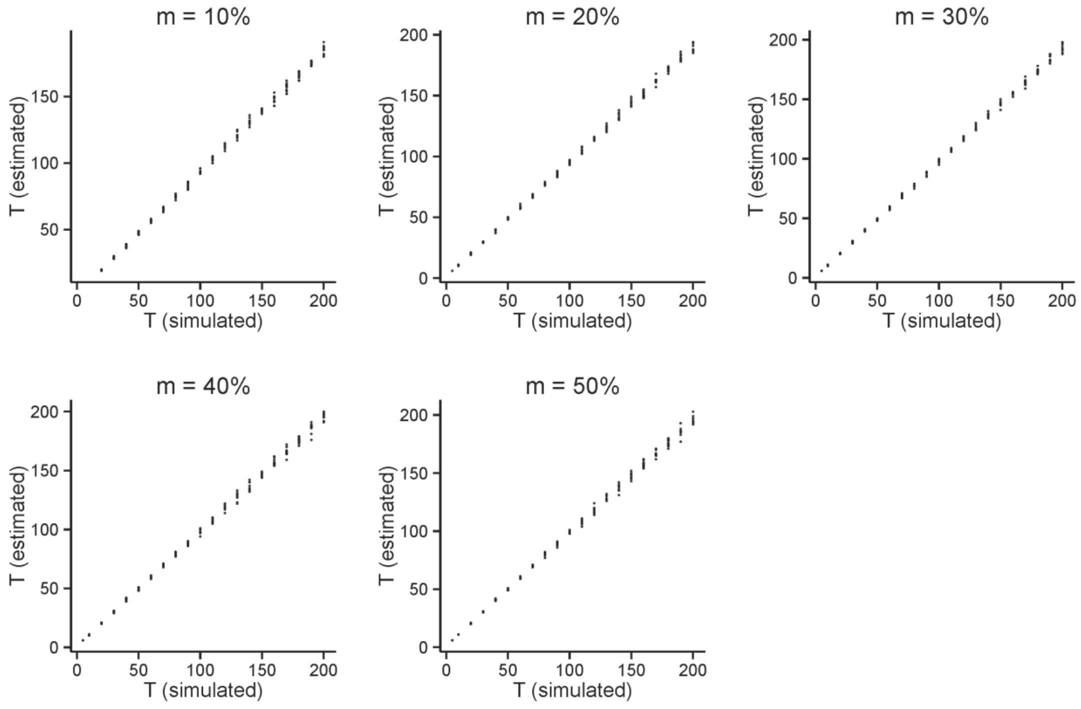

Fig. S6. Generation estimated for each simulation replicate under CGFR model.

Supplementary Figure S7

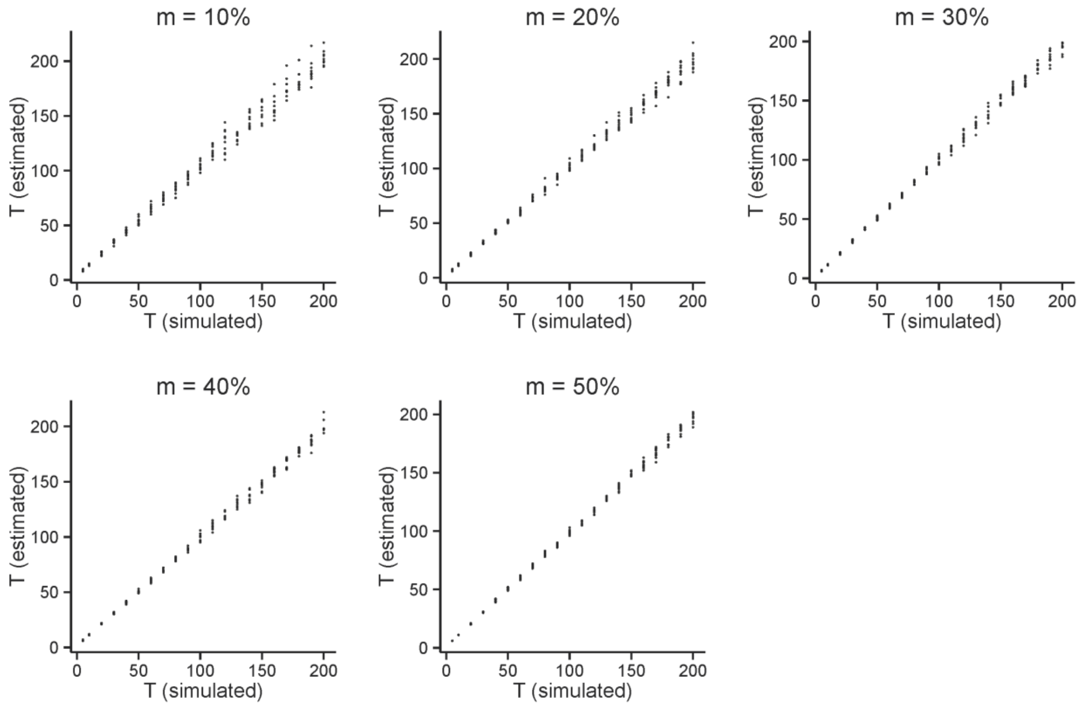

Fig. S7. Generation estimated for each simulation replicate under CGFD model.

Supplementary Figure S8

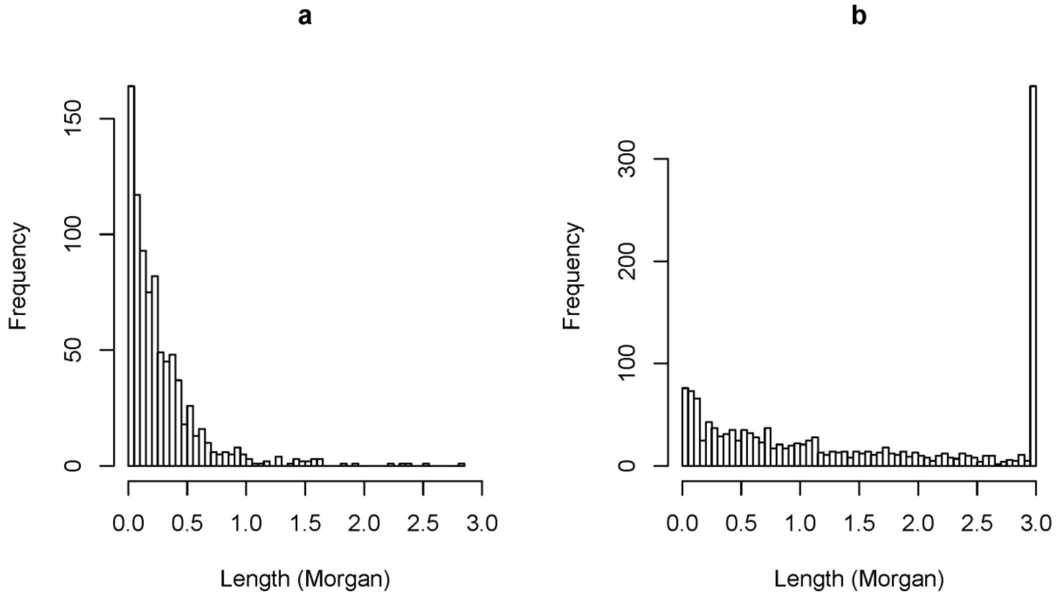

Fig. S8. **Simulated distributions of ancestral tracks in the case of incorrect determination. (a)** and **(b)** are the empirical distribution of ancestral tracks from population 1 and from population 2, respectively. The simulated model is GA model, admixture proportion and time is 10% and 5 generations, respectively.

Supplementary Figure S9

a

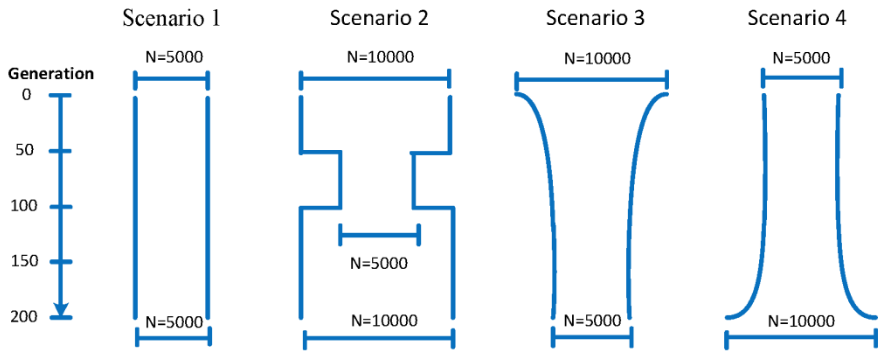

b

Model	Scenario 1	Scenario 2	Scenario 3	Scenario 4
HI	194	200	198	196
GA	196	196	194	191
CGF	201	196	194	198

Fig. S9. **Generation estimations under different demographic models. (a)**: Four demographic models. Scenario 1: Population with constant size; Scenario 2: Population with bottleneck between 50 and 100 generations; Scenario 3: Population with exponential size expansion; Scenario 3: Population with exponential size reduction. *N* is the effective population size. **(b)**: Generation estimations under these four Scenarios. The simulated admixture proportion and time is 50% and 200 generations, respectively.

Supplementary Table S1

Model (simulated)	T (simulated)	POP1	POP2	m (inferred)	Model (inferred)	T (inferred)	95%CI
HI	10	YRI	CEU	0.297787	HI (100%)	10	[10, 10]
HI	20	YRI	CEU	0.29644	HI (100%)	19.98	[18.95, 19.01]
HI	50	YRI	CEU	0.29517	HI (100%)	43.57	[43.47, 43.67]
HI	100	YRI	CEU	0.295788	GA (89%)	130.3	[130, 130.5]
GA	10	YRI	CEU	0.300604	GA (69%)	10.12	[10.04, 10.19]
GA	20	YRI	CEU	0.282173	GA (100%)	18.97	[18.92, 19.02]
GA	50	YRI	CEU	0.298265	GA (100%)	40.93	[40.82, 41.04]
GA	100	YRI	CEU	0.296194	GA (100%)	83.6	[83.43, 83.77]
CGFR	10	YRI	CEU	0.303632	CGFR (85%)	10.11	[10.04, 10.17]
CGFR	20	YRI	CEU	0.293441	CGFR (79%)	18.11	[18.04, 18.19]
CGFR	50	YRI	CEU	0.294307	CGFR (100%)	42.19	[42.08, 42.3]
CGFR	100	YRI	CEU	0.304212	CGFR (100%)	86.4	[86.24, 86.56]
CGFD	10	CEU	YRI	0.292857	CGFD (54%)	11.94	[11.86, 12.03]
CGFD	20	CEU	YRI	0.305231	CGFD (93%)	20.83	[20.75, 20.91]
CGFD	50	CEU	YRI	0.315461	CGFD (100%)	45.38	[45.26, 45.5]
CGFD	100	CEU	YRI	0.320606	CGFD (100%)	106.7	[106.5, 106.8]

Table S1. The estimation result of simulation tracks inferred by HAPMIX. Model (simulated): Simulated admixture model; T (simulated): Simulated admixture time; POP1: Reference population one; POP2: Reference population two; m (inferred):

inferred admixture proportion of POP1; Model (inferred): Inferred admixture model, percentage in the parenthesis is the support rate in 100 times bootstrapping; *T* (inferred): Inferred admixture time; 95%CI: 95% confidence interval of the estimated admixture time. The simulated proportion of POP1 is 30%.

Supplementary Text S1. Some detail produces of our method.

A. Detailed calculation for the length distribution of ancestral tracks under HI, GA and CGF models

For HI model (see Supplementary Fig. S2(a)), the ancestry proportions from population 1 and population 2 at generation t are

$$m_1(t) = \begin{cases} m, & t = 1 \\ 0, & 2 \le t \le T \end{cases} \text{ and } m_2(t) = \begin{cases} 1-m, & t = 1 \\ 0, & 2 \le t \le T \end{cases}.$$

From Equation (1) and (2), we can get

$$I(t) = \begin{cases} 0, & t = 1 \\ 1, & 2 \le t \le T \end{cases},$$

$$H_1(t) = m \text{ and } H_2(t) = 1-m, 1 \le t \le T.$$

Then we can easily get $s_i(t)$ from Equation (3) and $u_i(t)$ from Equation (4),

$$s_1(t) = \begin{cases} m, & t = 1 \\ 0, & 2 \le t \le T \end{cases}, s_2(t) = \begin{cases} (1-m), & t = 1 \\ 0, & 2 \le t \le T \end{cases};$$

and

$$u_1(t) = \begin{cases} (1-m)T, & t = 1 \\ 0, & 2 \le t \le T \end{cases}, u_2(t) = \begin{cases} mT, & t = 1 \\ 0, & 2 \le t \le T \end{cases}.$$

Substituting $u_i(t)$ and $s_i(t)$ into the Equation (5), we obtain the length distribution of ancestral tracks from population 1 and population 2 in HI model,

$$f_1(x; m, T) = (1-m)Te^{-(1-m)Tx},$$

and

$$f_2(x; m, T) = mTe^{-mTx}.$$

The conditional length distribution of ancestral tracks longer than a specific threshold C is as follows:

$$f_1(x; m, T|x > C) = (1-m)Te^{-(1-m)T(x-C)},$$

$$f_2(x; m, T|x > C) = mTe^{-mT(x-C)}.$$

We can also get the expectations and variances of the ancestral tracks from Equation (7) and Equation (8),

$$E(X_1) = \frac{1}{(1-m)T}, E(X_2) = \frac{1}{mT};$$
$$Var(X_1) = \frac{1}{(1-m)^2T^2}, Var(X_2) = \frac{1}{m^2T^2}.$$

For GA model (see Supplementary Fig. S2(b)), the ancestry proportions from population 1 and population 2 at generation t are

$$m_1(t) = \begin{cases} m, & t = 1 \\ m/T, & 2 \le t \le T \end{cases} \text{ and } m_2(t) = \begin{cases} 1-m, & t = 1 \\ (1-m)/T, & 2 \le t \le T \end{cases}.$$

Similarly, we can get $H_i(t)$, $s_i(t)$ and $u_i(t)$ as follows,

$$H_1(t) = m, H_2(t) = 1 - m;$$

and

$$s_1(t) = \begin{cases} m\left(1-\frac{1}{T}\right)^{T-1}, & t = 1 \\ \frac{m}{T}\left(1-\frac{1}{T}\right)^{T-t}, & 2 \le t \le T \end{cases},$$

$$s_2(t) = \begin{cases} (1-m)\left(1-\frac{1}{T}\right)^{T-1}, & t = 1 \\ \frac{1-m}{T}\left(1-\frac{1}{T}\right)^{T-t}, & 2 \le t \le T \end{cases};$$

and

$$u_1(t) = (T-t+1)(1-m), u_2(t) = (T-t+1)m.$$

The distributions of population 1 and population 2 in GA model are

$$f_1(x; m, T)$$
$$= (1-m)\left[\frac{T^2e^{-T(1-m)x} + \sum_{t=2}^{T}(T-t+1)^2\frac{1}{T}\left(1-\frac{1}{T}\right)^{1-t} e^{-(T-t+1)(1-m)x}}{T + \sum_{t=2}^{T}(T-t+1)\frac{1}{T}\left(1-\frac{1}{T}\right)^{1-t}}\right],$$

$$f_2(x;m,T) = m\left[\frac{T^2e^{-Tmx} + \sum_{t=2}^{T}(T-t+1)^2\frac{1}{T}\left(1-\frac{1}{T}\right)^{1-t}e^{-(T-t+1)mx}}{T+\sum_{t=2}^{T}(T-t+1)\frac{1}{T}\left(1-\frac{1}{T}\right)^{1-t}}\right].$$

The conditional length distribution of ancestral tracks longer than a specific threshold C is as follows:

$$f_1(x;m,T|x>C)$$
$$= \frac{(1-m)\left[T^2e^{-T(1-m)x} + \sum_{t=2}^{T}(T-t+1)^2\frac{1}{T}\left(1-\frac{1}{T}\right)^{1-t}e^{-(T-t+1)(1-m)x}\right]}{Te^{-T(1-m)C} + \sum_{t=2}^{T}(T-t+1)\frac{1}{T}\left(1-\frac{1}{T}\right)^{1-t}e^{-(T-t+1)(1-m)C}},$$

$$f_2(x;m,T|x>C) = \frac{m\left[T^2e^{-Tmx} + \sum_{t=2}^{T}(T-t+1)^2\frac{1}{T}\left(1-\frac{1}{T}\right)^{1-t}e^{-(T-t+1)mx}\right]}{Te^{-TmC} + \sum_{t=2}^{T}(T-t+1)\frac{1}{T}\left(1-\frac{1}{T}\right)^{1-t}e^{-(T-t+1)mC}}.$$

The expectations and variances of the ancestral tracks are as follows:

$$E(X_1) = \frac{T+\sum_{t=2}^{T}\left(1-\frac{1}{T}\right)^{1-t}}{(1-m)\left[T^2+\sum_{t=2}^{T}(T-t+1)\left(1-\frac{1}{T}\right)^{1-t}\right]},$$

$$E(X_2) = \frac{T+\sum_{t=2}^{T}\left(1-\frac{1}{T}\right)^{1-t}}{m\left[T^2+\sum_{t=2}^{T}(T-t+1)\left(1-\frac{1}{T}\right)^{1-t}\right]}.$$

$$Var(X_1) = \frac{1}{(1-m)^2}\left[\frac{2\left(1+\sum_{t=2}^{T}\frac{\left(1-\frac{1}{T}\right)^{1-t}}{T-t+1}\right)}{T^2+\sum_{t=2}^{T}(T-t+1)\left(1-\frac{1}{T}\right)^{1-t}} - \left(\frac{T+\sum_{t=2}^{T}\left(1-\frac{1}{T}\right)^{1-t}}{T^2+\sum_{t=2}^{T}(T-t+1)\left(1-\frac{1}{T}\right)^{1-t}}\right)^2\right],$$

$$Var(X_2) = \frac{1}{m^2}\left[\frac{2\left(1+\sum_{t=2}^{T}\frac{\left(1-\frac{1}{T}\right)^{1-t}}{T-t+1}\right)}{T^2+\sum_{t=2}^{T}(T-t+1)\left(1-\frac{1}{T}\right)^{1-t}} - \left(\frac{T+\sum_{t=2}^{T}\left(1-\frac{1}{T}\right)^{1-t}}{T^2+\sum_{t=2}^{T}(T-t+1)\left(1-\frac{1}{T}\right)^{1-t}}\right)^2\right].$$

For CGF model (see Supplementary Fig. S2(c)), the ancestry proportions from population 1 and population 2 at generation t are

$$m_1(t) = \begin{cases} 1-\alpha, & t=1 \\ 0, & 2 \le t \le T \end{cases} \text{ and } m_2(t) = \alpha, 1 \le t \le T.$$

where $\alpha = 1 - m^{1/T}$. Then

$$H_1(t) = (1-\alpha)^t, H_2(t) = 1-(1-\alpha)^t;$$

and

$$s_1(t) = \begin{cases} m, & t=1 \\ 0, & 2 \le t \le T' \end{cases}$$

$$s_2(t) = \alpha(1-\alpha)^{T-t} = \left(1-m^{1/T}\right)m^{(T-t)/T};$$

and

$$u_1(t) = \sum_{k=t}^{T}(1-(1-\alpha)^k) = (T-t+1) - \frac{m^{t/T} - m^{(T+1)/T}}{1-m^{1/T}},$$

$$u_2(t) = \sum_{k=t}^{T}(1-\alpha)^k = \frac{m^{t/T} - m^{(T+1)/T}}{1-m^{1/T}}.$$

Then the distribution of ancestral tracks in CGF model is as follows:

$$f_1(x;m,T) = \left(T - \frac{(1-m)m^{1/T}}{1-m^{1/T}}\right)e^{-\left(T-\frac{(1-m)m^{1/T}}{1-m^{1/T}}\right)x},$$

$$f_2(x;m,T) = \frac{\sum_{t=1}^{T} m^{-t/T}\left(m^{t/T} - m^{(T+1)/T}\right)^2 e^{-\left(\frac{m^{t/T}-m^{(T+1)/T}}{1-m^{1/T}}\right)x}}{\sum_{t=1}^{T}(1-m^{(T+1-t)/T})(1-m^{1/T})}.$$

The conditional length distribution of ancestral tracks longer than a specific threshold

C is as follows:

$$f_1(x;m,T|x>C) = \left(T - \frac{(1-m)m^{1/T}}{1-m^{1/T}}\right)e^{-\left(T-\frac{(1-m)m^{\frac{1}{T}}}{1-m^{\frac{1}{T}}}\right)(x-C)},$$

$$f_2(x;m,T) = \frac{(1-m^{1/T})\sum_{t=1}^{T} m^{-t/T}\left(m^{t/T} - m^{(T+1)/T}\right)^2 e^{-\left(\frac{m^{t/T}-m^{(T+1)/T}}{1-m^{1/T}}\right)x}}{\sum_{t=1}^{T} m^{-t/T}(m^{t/T} - m^{(T+1)/T})e^{-\left(\frac{m^{t/T}-m^{(T+1)/T}}{1-m^{1/T}}\right)C}}.$$

The expectations and variances of the ancestral tracks are as follows:

$$E(X_1) = \frac{(1-m^{1/T})}{T(1-m^{1/T}) - (1-m)m^{1/T}},$$

$$E(X_2) = \frac{(1-m)}{m}\frac{(1-m^{1/T})}{T(1-m^{1/T})-(1-m)m^{1/T}};$$

$$Var(X_1) = \left(\frac{(1-m^{1/T})}{T(1-m^{1/T})-(1-m)m^{1/T}}\right)^2,$$

$$Var(X_2) = \frac{2\left(1-m^{1/T}\right)^3 \sum_{t=1}^{T}\frac{m^{-t/T}}{m^{t/T}-m^{(T+1)/T}}}{T(1-m^{1/T})-(1-m)m^{1/T}} - \left(\frac{(1-m)(1-m^{1/T})}{mT(1-m^{1/T})-m(1-m)m^{1/T}}\right)^2.$$

B. The proof of Equation (17)

When the number of the ancestry populations K is 2, Equation (17) is always true.

Proof: In order to prove

$$\frac{E(X_1)}{E(X_2)} = \frac{m}{1-m},$$

we need to prove

$$\frac{\frac{\sum_{t=1}^{T} s_1(t)}{\sum_{t=1}^{T} u_1(t)s_1(t)}}{\frac{\sum_{t=1}^{T} s_2(t)}{\sum_{t=1}^{T} u_2(t)s_2(t)}} = \frac{m}{1-m}.$$

We note that $\sum_{t=1}^{T} s_1(t) = m$ and $\sum_{t=1}^{T} s_2(t) = 1-m$, thus the problem turns into proving

$$\sum_{t=1}^{T} u_1(t)s_1(t) = \sum_{t=1}^{T} u_2(t)s_2(t).$$

Substituting Equation (3) and Equation (4) into the above Equation, we can get

$$\begin{aligned}\sum_{t=1}^{T} u_1(t)s_1(t) &= \sum_{t=1}^{T}\sum_{k=t}^{T}(1 - H_1(k))\, m_1(t)\left(\prod_{l=t+1}^{T} I(l)\right)\\ &= \sum_{t=1}^{T}\sum_{k=t}^{T} H_2(k)\, m_1(t)\left(\prod_{l=t+1}^{T} I(l)\right)\\ &= \sum_{k=1}^{T}\sum_{t=1}^{k} H_2(k)\, m_1(t)\left(\prod_{l=t+1}^{T} I(l)\right)\\ &= \sum_{k=1}^{T}\sum_{s=1}^{k} m_2(s)\left(\prod_{l=s+1}^{k} I(l)\right)\sum_{t=1}^{k} m_1(t)\left(\prod_{l=t+1}^{T} I(l)\right)\\ &= \sum_{k=1}^{T}\sum_{s=1}^{k} H_1(k)\, m_2(s)\left(\prod_{l=s+1}^{T} I(l)\right) = \sum_{t=1}^{T} u_2(t)s_2(t).\end{aligned}$$

C. Detail of Bootstrapping Procedures

Let n be the number of bootstrapping, p be the proportion of tracks sampled in each bootstrapping repeat, α be the significance level to compute confidence interval, and N_i be the total number of tracks from *i-th* ancestral population.

1) For each bootstrapping replicate, we sample pN_i tracks from *i-th* ancestral population, and combine them as the new dataset to select the optimal model and estimate the corresponding admixture time with the procedures described in Materials and Methods;

2) At the end of bootstrapping, the optimal model of the highest number of occurrence (o) is chosen as the bootstrapped optimal model, then the supporting rate is $\frac{o}{n} \times 100\%$;

3) We define a set A as all the admixture times under the bootstrapped optimal model, then we regard the mean time $\bar{T}$ as the bootstrapped estimator of the admixture time,

$$\bar{T} = \frac{1}{|A|}\sum_{T\in A} T,$$

where $|A|$ is the number of elements of A.

4) Since the variance is unknown, we calculate the confidence interval of the admixture time with significance level α as:

$$\left[\bar{T} - \frac{S}{\sqrt{|A|}} t_{\alpha/2}(|A| - 1), \bar{T} + \frac{S}{\sqrt{|A|}} t_{\alpha/2}(|A| - 1)\right],$$

where $S^2 = \frac{1}{|A|-1} \sum_{T \in A} (T - \bar{T})^2$.

Supplementary Data Legends

Data S1. Detailed parameters of admixture model for simulation 1. Sheet 1: Simulation 1A; Sheet 2: Simulation 1B; Sheet 3: Simulation 1C; Sheet 4: Simulation 1D.

Data S2. Details of parameters estimation and model selection of 1050 simulation replicates under HI model. Sheet 1: The summary of time estimated of all cases. Sheet 1: Admixture proportion is 10%; Sheet 2: Admixture proportion is 20%; Sheet 3: Admixture proportion is 30%; Sheet 4: Admixture proportion is 40%; Sheet 5: Admixture proportion is 50%.

Data S3. Details of parameters estimation and model selection of 1050 simulation replicates under GA model. Sheet 1: The summary of time estimated of all cases. Sheet 1: Admixture proportion is 10%; Sheet 2: Admixture proportion is 20%; Sheet 3: Admixture proportion is 30%; Sheet 4: Admixture proportion is 40%; Sheet 5: Admixture proportion is 50%.

Data S4. Details of parameters estimation and model selection of 1050 simulation replicates under CGF (population 1 as recipient) model. Sheet 1: The summary of time estimated of all cases. Sheet 1: Admixture proportion is 10%; Sheet 2: Admixture proportion is 20%; Sheet 3: Admixture proportion is 30%; Sheet 4: Admixture proportion is 40%; Sheet 5: Admixture proportion is 50%.

Data S5. Details of parameters estimation and model selection of 1050 simulation replicates under CGF (population 1 as donor) model. Sheet 1: The summary of

time estimated of all cases. Sheet 1: Admixture proportion is 10%; Sheet 2: Admixture proportion is 20%; Sheet 3: Admixture proportion is 30%; Sheet 4: Admixture proportion is 40%; Sheet 5: Admixture proportion is 50%.

Data S6. Summary of simulations whose model was incorrectly determined. Model (simulated) and Model (inferred) are the simulated and inferred admixture model, respectively. m (simulated) and m (inferred) are the simulated and inferred admixture proportion, respectively. T (simulated) and T (inferred) the simulated and inferred admixture time, respectively.

Data S7. The probability p of ancestral tracks whose length are longer than a specific threshold C under different admixture time and proportions. Sheet 1: C=0.5cM; Sheet 2: C=1cM; Sheet 3: C=1.5cM; Sheet 4: C=2cM. T: the admixture time; m: the admixture proportion; p: the the probability of ancestral tracks larger than a specific threshold C.
